# Severe symptomatic nickel allergy following stent graft implantation requiring excision and external iliac artery reconstruction

**DOI:** 10.1016/j.jvscit.2022.08.013

**Published:** 2022-08-30

**Authors:** Rhusheet Patel, Wesley Moore, Juan Carlos Jimenez

**Affiliations:** a Division of Vascular and Endovascular Surgery, Gonda Vascular Center, University of Texas Southwestern Medical Center, Dallas, TX; b Division of Vascular and Endovascular Surgery, Gonda (Goldschmied) Vascular Center, David Geffen School of Medicine at UCLA, Los Angeles, CA

**Keywords:** Nickel Allergy, Nitinol, Stent-graft, Endovascular

## Abstract

Although nickel allergy is a common cause of contact dermatitis, systemic reactions to nitinol stents are rare. A 61-year-old woman had presented with a nonhealing toe wound. Angiography revealed an external iliac artery stenosis, which was treated with a nitinol stent graft. However, she developed severe truncal pruritus, and within 3 months, her external iliac stent graft had thrombosed. Allergy testing revealed nickel sensitivity. After medical therapy had failed, stent graft removal was performed, resulting in complete resolution of her symptoms. The present case demonstrates a rare allergic reaction to the nitinol in commercially available stent grafts. Pruritus and rash are rare reactions to stenting; however, a nitinol allergy should be considered for patients with no other identifiable primary source.

Nitinol is an alloy composed of 55% nickel and 45% titanium. Its unique physical properties make it an ideal scaffold for self-expanding peripheral stents. Although nickel allergy causing contact dermatitis is present in as many as 18% of the population, systemic reactions to nickel alloy stents have rarely been reported.^1^ We present a case of systemic pruritus and stent graft thrombosis requiring excision and bypass after placement of a Viabahn stent graft (Gore Medical, Flagstaff, AZ) in the external iliac artery. The patient provided written informed consent for the report of her case details and imaging studies.

## Case report

A 61-year-old woman had initially presented to a community hospital with a nonhealing left toe wound. She denied claudication or ischemic rest pain. She had a medical history significant for hypertension and dyslipidemia. The patient had no prior reported history of a nickel allergy. She had no other implants containing nickel or any material predisposing to this reaction. After noninvasive testing, the patient underwent left lower extremity angiography. An external iliac artery stenosis was treated with a self-expanding, covered, 7-mm × 7.5-cm Viabahn nitinol stent graft (Gore Medical). Based on magnetic resonance angiography from the outside facility, the common iliac artery proximal to the stent graft measured 7 mm in diameter and the distal external iliac artery measured 6 to 7 mm. She was instructed to take aspirin 81 mg daily after her procedure. However, the patient had presented to our institution 3 months later with a severe, widespread truncal rash and pruritus, which had begun 2 days after stent graft placement and had been refractory to topical (triamcinolone cream) and oral antihistamine (cetirizine) treatment for 3 months, interfering with her daily activities.

Arterial duplex ultrasound from her outside facility demonstrated an occluded stent with monophasic signals at the common femoral artery. Stent graft occlusion was confirmed by magnetic resonance angiography, with distal reconstitution and three-vessel runoff. The ankle brachial index performed at our office was 0.98 on the affected left side compared with 1.3 on the right. Her flow signals distal to the occluded stent graft were dampened compared with those to her right foot. The patient underwent patch testing to nickel sulfate-hexahydrate by an immunology specialist, which revealed a nickel allergy.

**Fig 1:**
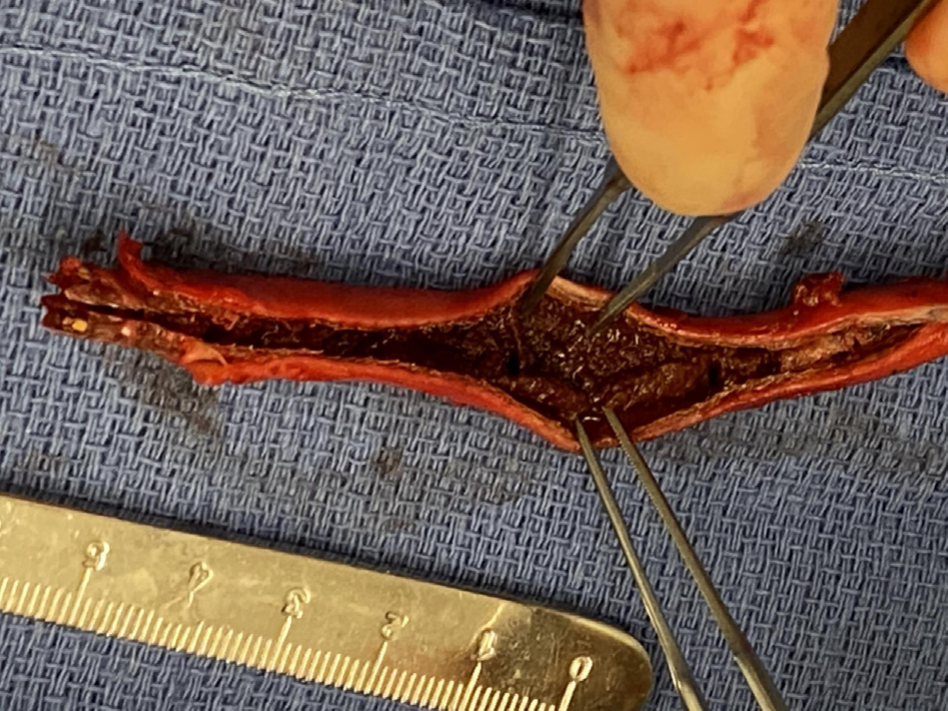
The external iliac artery and stent graft were completely excised. Acute stent graft thrombosis was present with no gross evidence of inflammatory changes surrounding the artery.

**Fig 2:**
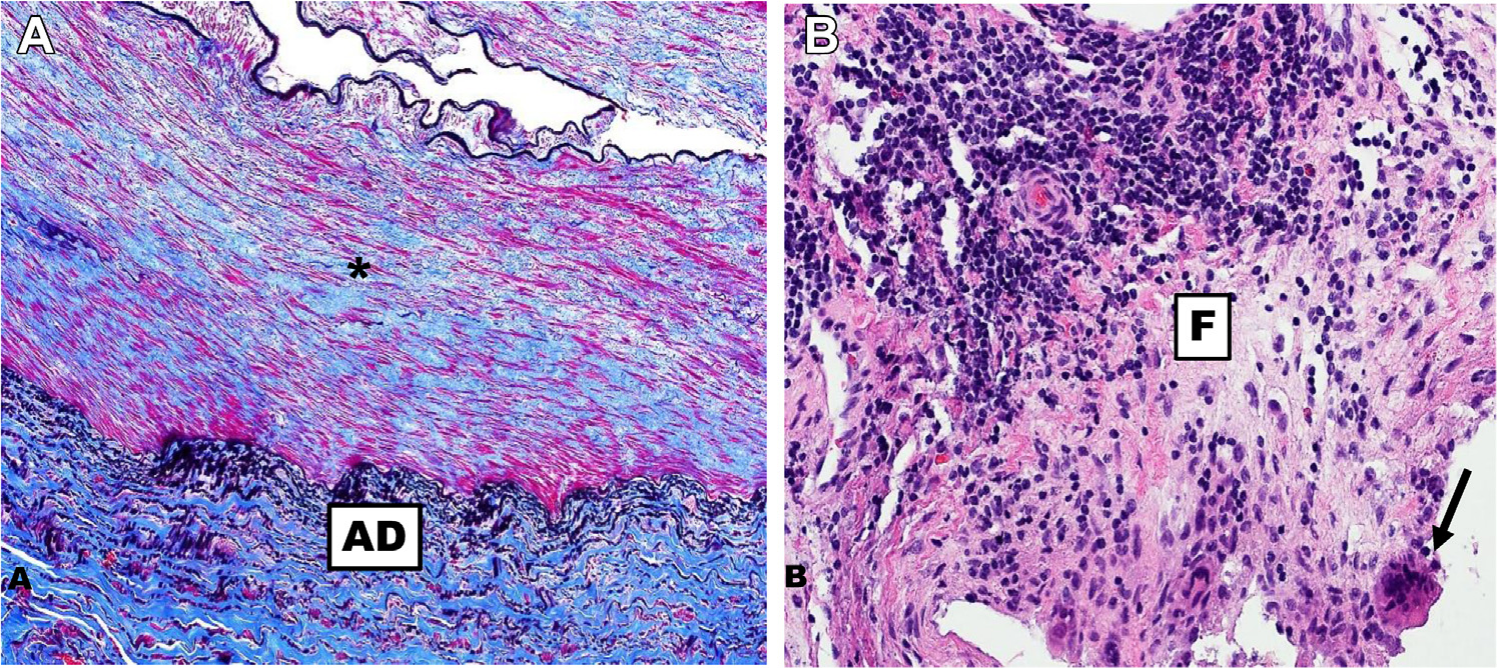
**A,** Arterial wall with loss of smooth muscle and fibrosis of both the media (*asterisk*) and adventitia (AD). **B,** Foreign body giant cells (*arrow*), chronic inflammation, and fibrosis (F), with rare eosinophils were present.

Given the patient’s severe symptoms, stent graft excision and arterial reconstruction were performed. An oblique suprainguinal incision and a left retroperitoneal approach exposed the left external iliac artery. Inflow was controlled proximally at the common iliac artery and left hypogastric artery. A separate oblique groin incision exposed the common femoral artery below the inguinal ligament. We clamped the common iliac artery proximally and the common femoral artery distally, and a longitudinal arteriotomy was made across the external iliac artery and stent graft. Because the stent graft was firmly incorporated, the external iliac artery was resected and replaced with an 8-mm Dacron interposition graft (Fig 1). Arterial pathologic examination demonstrated chronic thrombus and fibrosis surrounding the stent graft. Reactionary foreign body giant cells and rare eosinophils were also noted within the vessel wall (Fig 2, *A* *and* *B*).

Postoperatively, the patient recovered without complications. Before discharge on postoperative day 2, she had had complete resolution of her truncal rash and pruritic symptoms, which continued through her outpatient follow-up. At her 1-month postoperative visit, she had continued resolution of her toe wound with a normal ankle brachial index. At her recent 1-year follow-up examination, she remained symptom free with an ankle brachial index of 1.17 on the right and 1.16 on the left.

## Discussion

Allergic symptoms resulting from peripheral vascular stenting remain rare, and few case reports have described these reactions during the past 10 years.^2^, ^3^, ^4^ The previous reports have similarly described patients who had presented with an intensely pruritic rash within 1 month of stent implantation. The spectrum of severity can range from localized eczematous dermatitis to a whole body desquamation, macular papular rash.^4^ These dermatologic reactions will often be refractory to topical therapies and systemic corticosteroids.

Patients with a history of nickel allergy who possibly will require stent placement should undergo consultation and testing with allergy experts to confirm a nickel sensitivity and exclude other possible iatrogenic or environmental causes. Metal sensitization can persist for years, and even a remote exposure can predispose patients to an implant reaction.^5^ Patch testing and skin biopsy could be warranted as a part of the evaluation. The cause of the systemic dermatologic symptoms is not entirely understood but has been thought to be secondary to a delayed type IV hypersensitivity reaction—an initial local immune response to a foreign antigen sensitizes immune T cells, which, in turn, release systemic cytokines, resulting in tissue damage.^6^^,^^7^ Although nickel can be a cause of allergic dermatitis and is the predominant component of nitinol, the amount of nickel ion released from nitinol is relatively small compared with other studied alloys.^8^ This likely explains the rarity of this presentation, even as the number of peripheral stenting cases has increased. Stainless steel and cobalt alloys used in balloon expandable and self-expanding peripheral stents also contain between 10% and 15% nickel. Thus, these could also be the cause of the systemic hypersensitivity reactions reported in the literature.^9^

The indication for stent resection in our case was the patient’s systemic allergic symptoms; however, stent thrombosis and inferior patency have also been associated with a nickel allergy. Described in the coronary literature, the mechanism is thought to be mediated through ICAM-1 (intracellular adhesion molecule-1). However, no mechanism has been definitively identified that explains this relationship. Present in the endothelium, ICAM-1 is a known inflammatory catalyst, and its expression is promoted by nickel ions.^10^ Prior reports have demonstrated a relationship between nickel allergy and coronary stent thrombosis; however, no causal relationship has ever been shown.^11^^,^^12^

Although the etiology of stent-related nickel allergy is not entirely clear, management for patients with severe symptoms refractory to treatment will require surgical resection and revascularization. Our patient experienced complete resolution of her symptoms after complete stent excision. The low incidence of nitinol stent allergy likely does not warrant a preoperative allergic evaluation for all patients. However, for patients with a known nickel allergy, the stent composition and the patient’s history should be critically evaluated before selecting a treatment plan.

## Conclusions

The present case has demonstrated a rare allergic reaction to the nitinol components of commercially available stent grafts. Complete stent excision and revascularization can be required for symptom resolution.

## References

[1] Thyssen J.P., Linneberg A., Menné T., Johansen J.D. (2007). The epidemiology of contact allergy in the general population—prevalence and main findings. Contact Dermatitis.

[2] Jetty P., Jayaram S., Veinot J., Pratt M. (2013). Superficial femoral artery nitinol stent in a patient with nickel allergy. J Vasc Surg.

[3] D'Arrigo G., Giaquinta A., Virgilio C., Davì A., Pierfrancesco V., Veroux M. (2014). Nickel allergy in a patient with a nitinol stent in the superficial femoral artery. J Vasc Interv Radiol.

[4] Guerra A., Kirkwood M. (2017). Severe generalized dermatitis in a nickel-allergic patient with a popliteal artery nitinol stent. J Vasc Surg Cases Innov Tech.

[5] Nguyen S.H., Dang T.P., MacPherson C., Maibach H., Maibach H.I. (2008). Prevalence of patch test results from 1970 to 2002 in a multi-centre population in North America (NACDG). Contact Dermatitis.

[6] Svedman C., Ekqvist S., Möller H., Björk J., Pripp C.M., Gruvberger B. (2009). A correlation found between contact allergy to stent material and restenosis of the coronary arteries. Contact Dermatitis.

[7] Aliağaoğlu C., Turan H., Erden I., Albayrak H., Ozhan H., Başar C. (2012). Relation of nickel allergy with in-stent restenosis in patients treated with cobalt chromium stents. Ann Dermatol.

[8] Honari G., Ellis S.G., Wilkoff B.L., Aronica M.A., Svensson L.G., Taylor J.S. (2008). Hypersensitivity reactions associated with endovascular devices. Contact Dermatitis.

[9] Univers J., Long C., Tonks S.A., Freeman M.B. (2018). Systemic hypersensitivity reaction to endovascular stainless steel stent. J Vasc Surg.

[10] Messer R.L., Wataha J.C., Lewis J.B., Lockwood L.E., Caughman G.B., Tseng W.Y. (2005). Effect of vascular stent alloys on expression of cellular adhesion molecules by endothelial cells. J Long Term Eff Med Implants.

[11] Norgaz T., Hobikoglu G., Serdar Z.A., Aksu H., Alper A.T., Ozer O. (2005). Is there a link between nickel allergy and coronary stent restenosis?. Tohoku J Exp Med.

[12] El-Mawardy R., Fuad H., Abdel-Salam Z., Ghazy M., Nammas W. (2011). Does nickel allergy play a role in the development of in-stent restenosis?. Eur Rev Med Pharmacol Sci.

